# Supplementary material for: Social inheritance can explain the structure of animal social networks
Source: Nat Commun. 2016 Jun 28;7:12084. doi: 10.1038/ncomms12084 (PMC4931239; doi:10.1038/ncomms12084)
Supplement: Supplementary Information — Supplementary Figures 1-14 and Supplementary Table 1 [file ncomms12084-s1.pdf]

Supplementary Figures

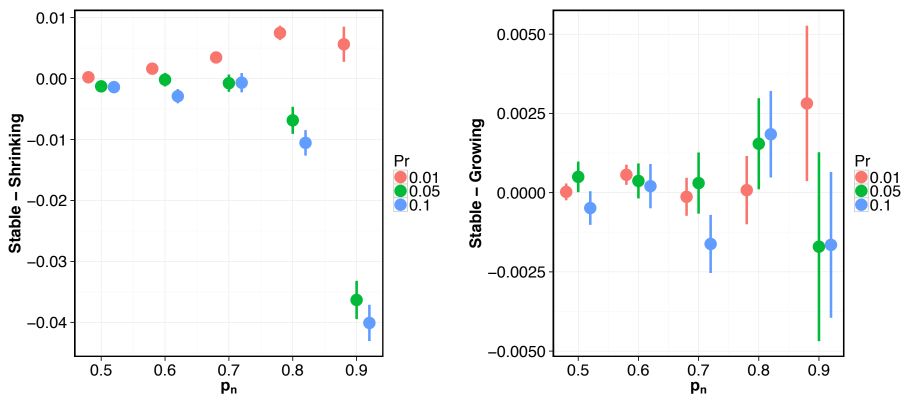

Supplementary Figure 1: **Density of growing and shrinking networks.** The difference in network density of simulated networks from our model between stable and shrinking (left) or growing (right) networks. Points and lines represent the mean difference and standard error, respectively. Red, green, and blue depict p_r values of 0.01, 0.05, and 0.1, respectively.

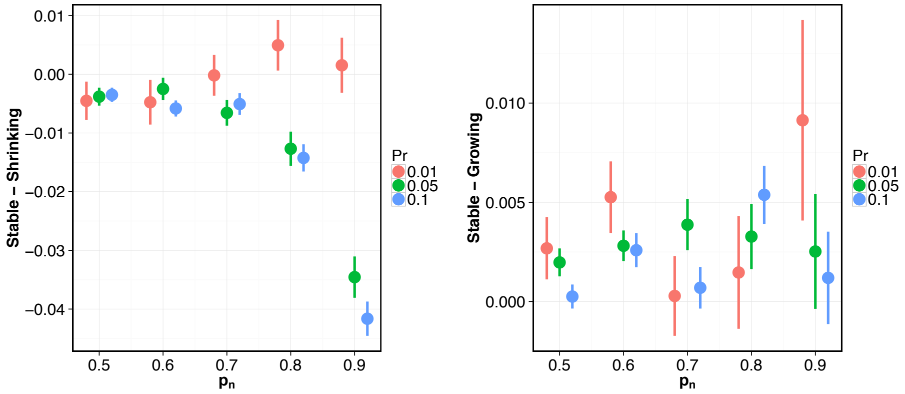

Supplementary Figure 2: **Clustering coefficients of growing and shrinking networks.** The difference in global clustering coefficient of simulated networks from our model between stable and shrinking (left) or growing (right) networks. Points and lines represent the mean difference and standard error, respectively. Red, green, and blue depict p_r values of 0.01, 0.05, and 0.1, respectively.

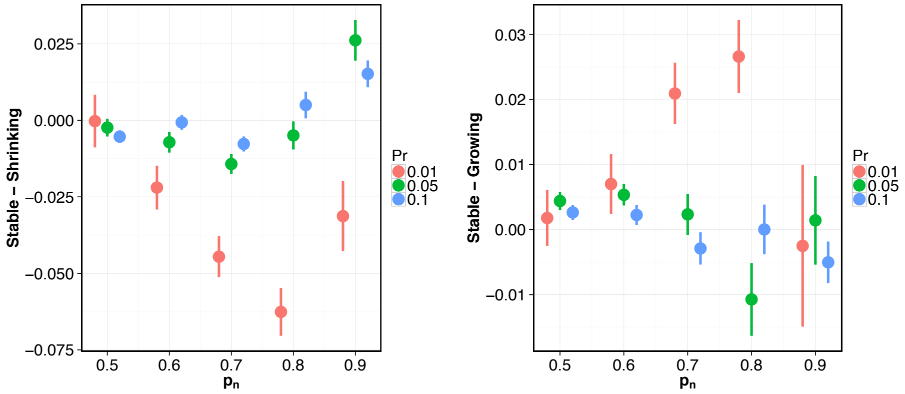

Supplementary Figure 3: **Modularity of growing and shrinking networks.** The difference in network modularity of simulated networks from our model between stable and shrinking (left) or growing (right) networks. Points and lines represent the mean difference and standard error, respectively. Red, green, and blue depict p_r values of 0.01, 0.05, and 0.1, respectively.

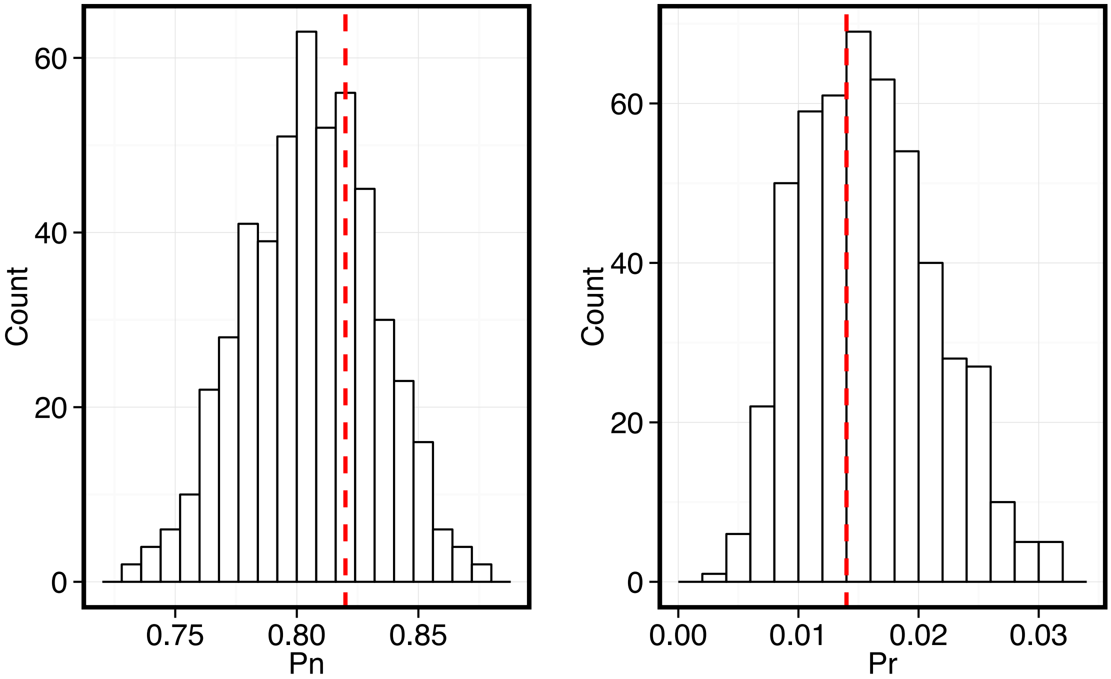

Supplementary Figure 4: **Example of PLS estimation using simulated network.** The distributions of predicted p_n (left) and p_r (right) values for 500 networks simulated using $p_n = 0.82, p_r = 0.014$ are plotted, along with the real parameter values (red dashed line).

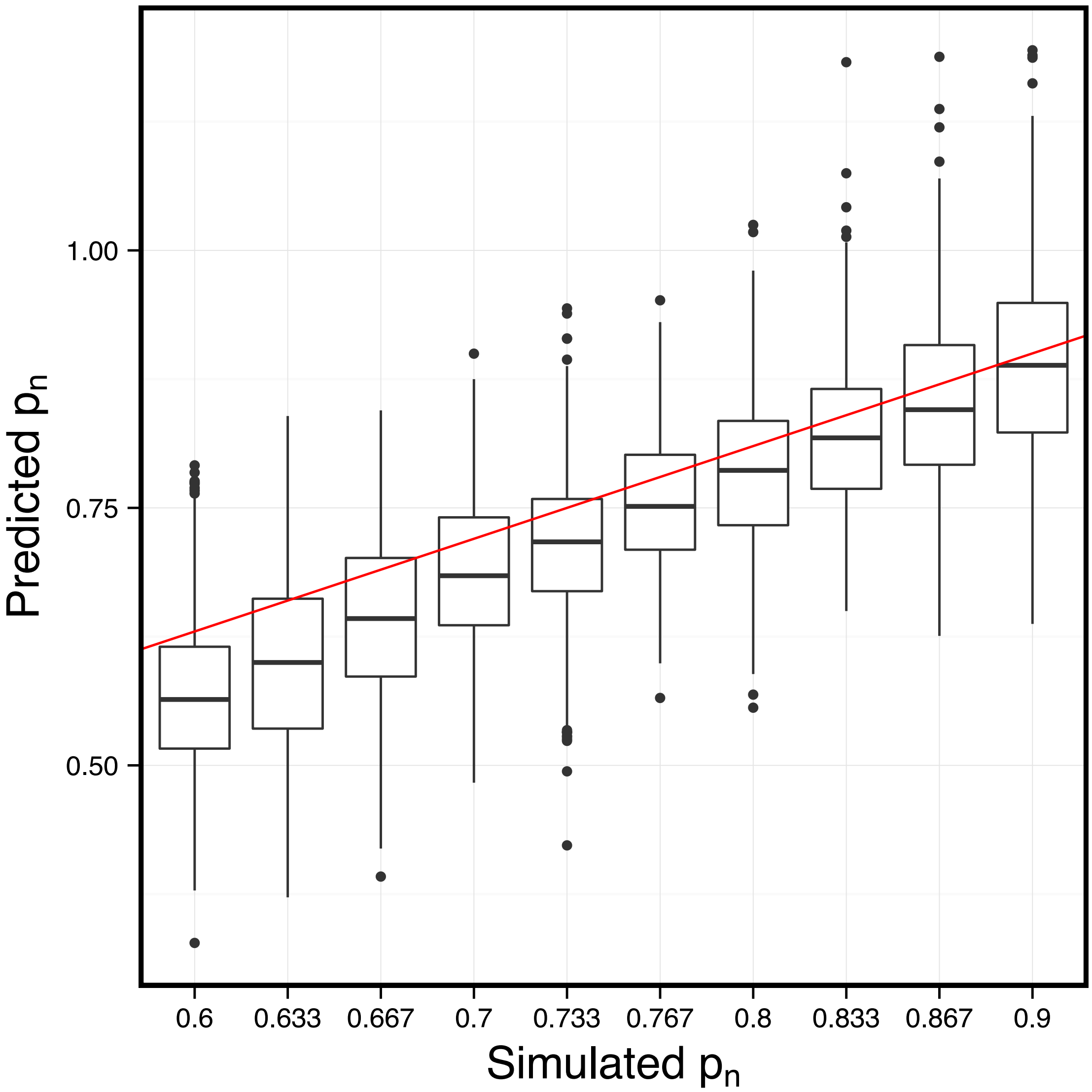

Supplementary Figure 5: **The PLS method estimates parameters with reasonable accuracy.**Distributions of predicted p_n values (box plot), compared to simulated values (red line). The predictions were generated after fitting a PLS regression to degrees and clustering coefficients of simulated networks. Thus, it is possible to predict the model parameter values when given an observed network. The bottom and top of the box mark the first and third quartiles. The upper whisker extends from the hinge to the highest value that is within 1.5*IQR of the hinge, where IQR is the inter-quartile range, or distance between the first and third quartiles. The lower whisker extends from the hinge to the lowest value within 1.5*IQR of the hinge. Data beyond the end of the whiskers are outliers and plotted as points.

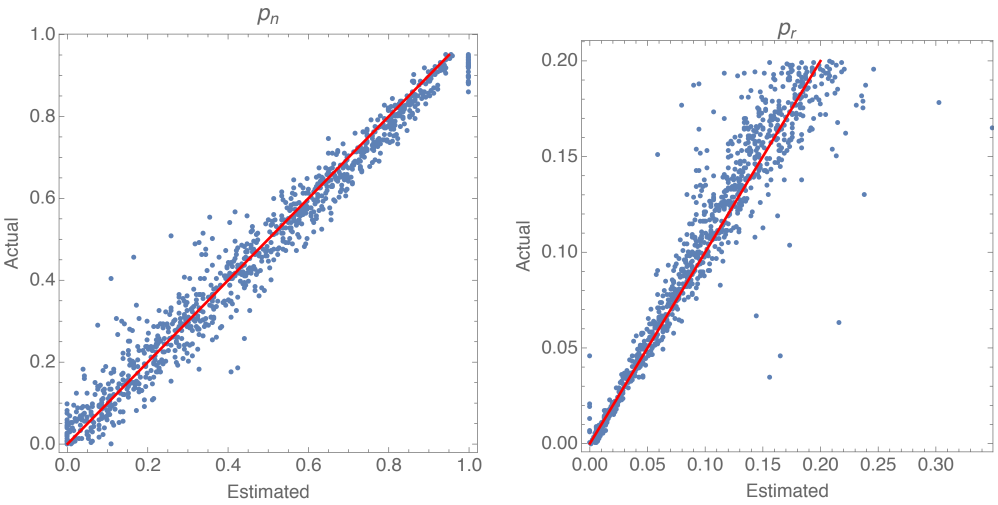

Supplementary Figure 6: **Estimation of model parameters using the analytical approximation.** The analytical approximations for the mean degree and clustering coefficient allow us to estimate network parameters with good accuracy. The two panels show for 1000 simulated networks ($N = 100$) the actual p_n (left) and p_r (right) values plotted against the values estimated from our analytical approximation. In each panel, the red line depicts the 1:1 relationship. For each simulation, the p_n and p_r values were drawn randomly from a uniform distribution on $[0, 0.95]$ and $[0, 0.2]$, respectively. We initialized each simulation with a random network and ran it for 2000 steps. We calculated the mean degree and local clustering coefficients for the resulting network. We used these values to numerically solve equations (2) and (13) for p_n and p_r, to obtain the estimates.

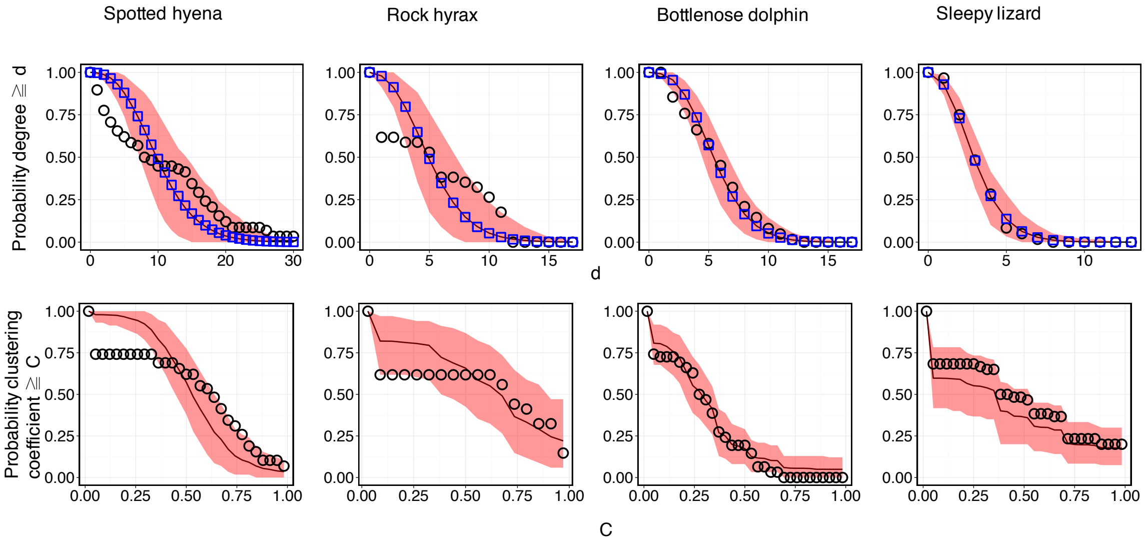

Supplementary Figure 7: **Fitting model to data using the analytical approximation.** This figure compares networks generated using parameters estimated from the analytical approximation to the data from four species. Upper row: Cumulative degree distributions of observed and simulated networks. Lower row: Cumulative clustering coefficient distributions of observed and simulated networks. Black circles represent observed values. Blue squares in the upper row depict mean-field estimation for the degree distribution. Red line notes mean values for 500 simulated networks (2000 simulation steps) with the same species-specific p_n and p_r values (given in Table 1), whereas light red area depicts 95% confidence intervals.

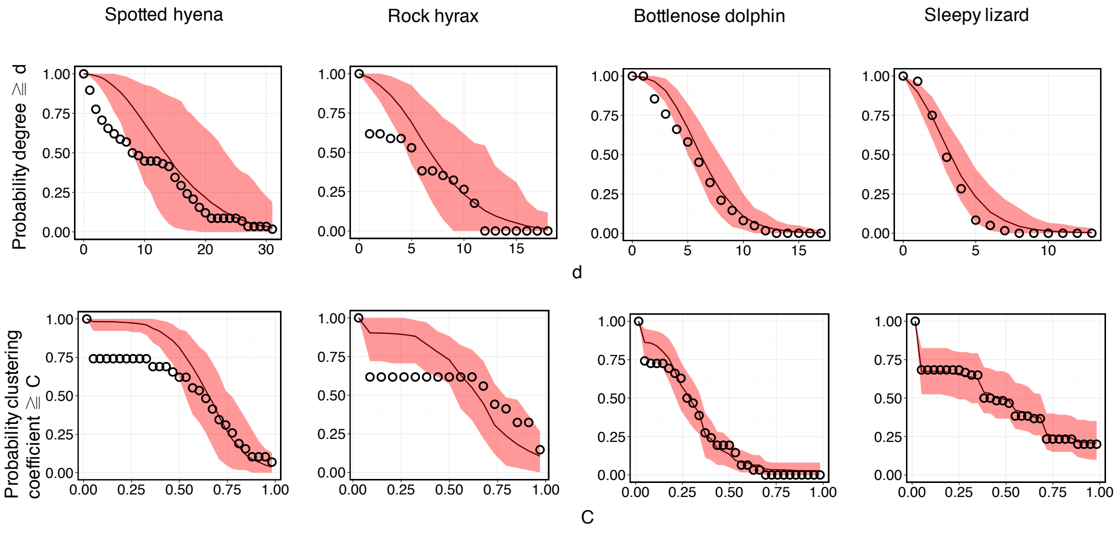

Supplementary Figure 8: **Comparing the two sex model output to networks of four species.** Upper row: Cumulative degree distributions of observed and simulated networks. Lower row: Cumulative clustering coefficient distributions of observed and simulated networks. Black circles represent observed values. Red line notes mean values for 500 simulated networks (2000 simulation steps) with the same species-specific p_n and p_r values (given in Table 1), whereas light red area depicts 95% confidence intervals.

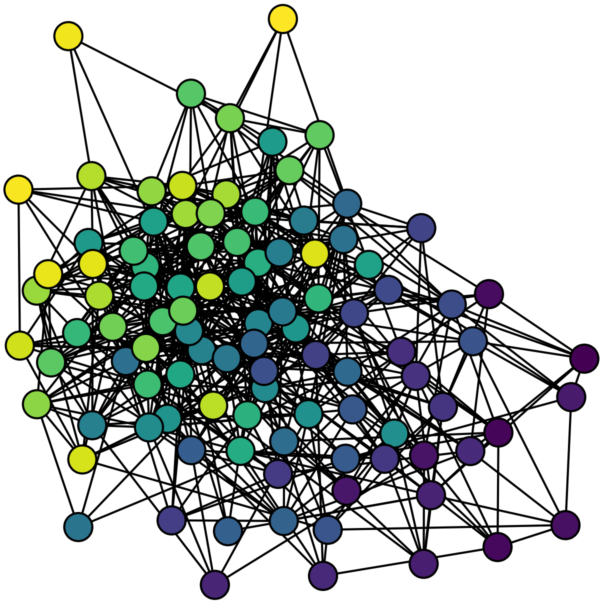

Supplementary Figure 9: **Example network generated with the assortative attachment model.** An example of a social network resulting from the explicit assortativity model, in which newborns are more likely to connect with similar individuals. Colors represent the values of an arbitrary trait, considered when forming bonds. See text for model definition and simulation parameters.

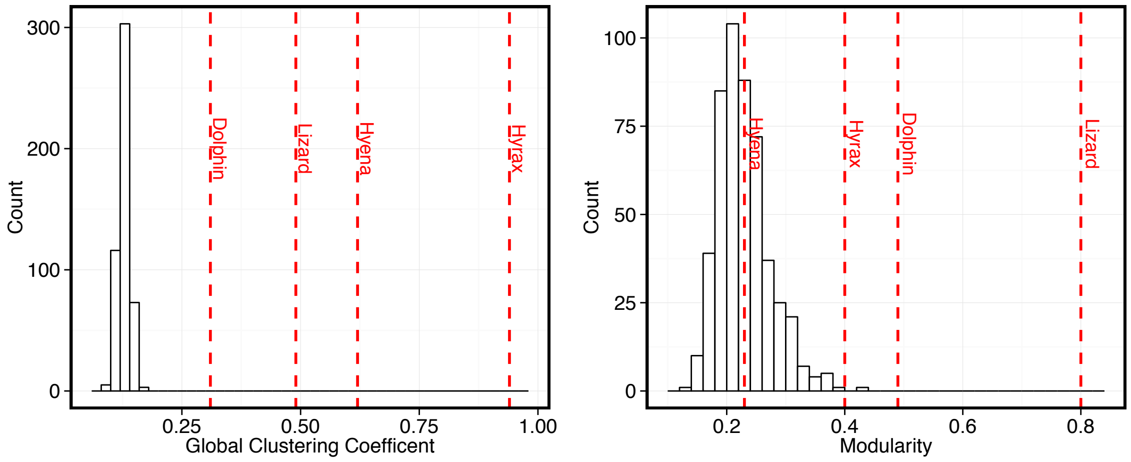

Supplementary Figure 10: **Clustering and modularity in the assortative attachment model.** A comparison of the global clustering coefficient and modularity of 500 networks resulting from the explicit assortativity model (see text for details) to the values of observed networks of four species. Distributions show value of network measures for model networks. Red line show values for observed networks. The global clustering coefficient of all model networks is much lower than that of observed networks. Similarly, the modularity of model networks is lower than observed networks, except for the spotted hyena.

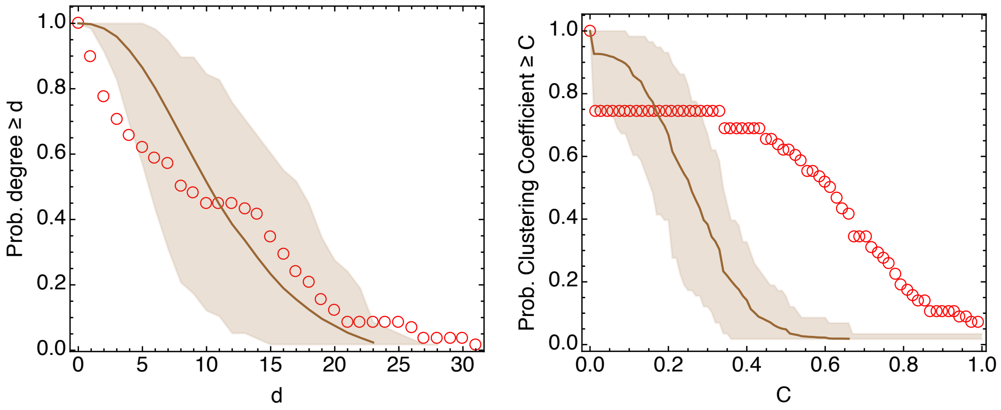

Supplementary Figure 11: Generalized preference model does not produce enough clustering. Degree (left) and local clustering (right) distributions obtained from the generalized preference model described in section , compared with the spotted hyena network from 1997 (red open circles). The brown solid line gives the mean of the preferential association model with $N = 58$, $k = 12$ and $\sigma = 0.05$, and the shaded region the 10th and 90th percentiles in each panel.

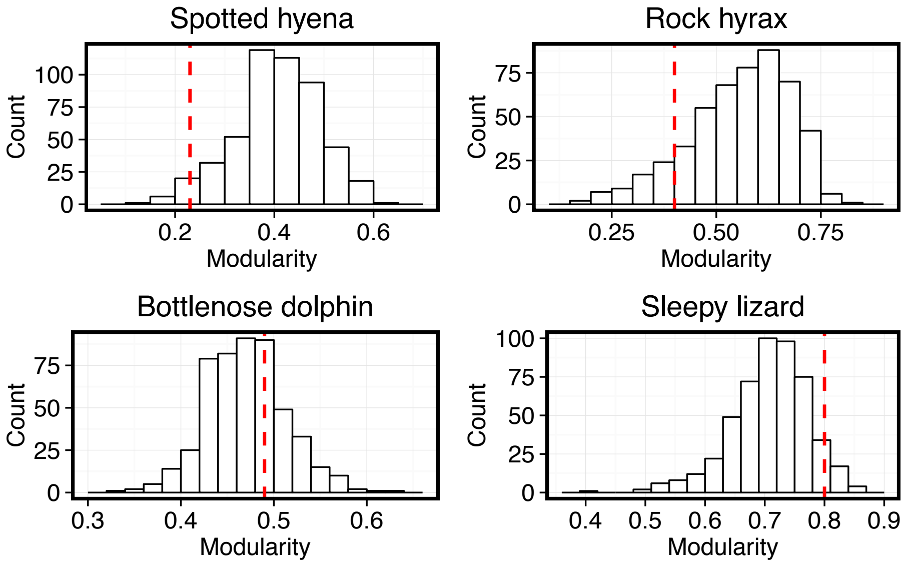

Supplementary Figure 12: **Modularity of networks with fitted model parameters.** The network modularity of simulated networks from our model (distribution), compared to modularity of observed networks (red line). Modularity was calculated after partitioning the network to communities using the Walktrap algorithm. In all four species, the observed modularity could be generated by the model, i.e. was not an outlier.

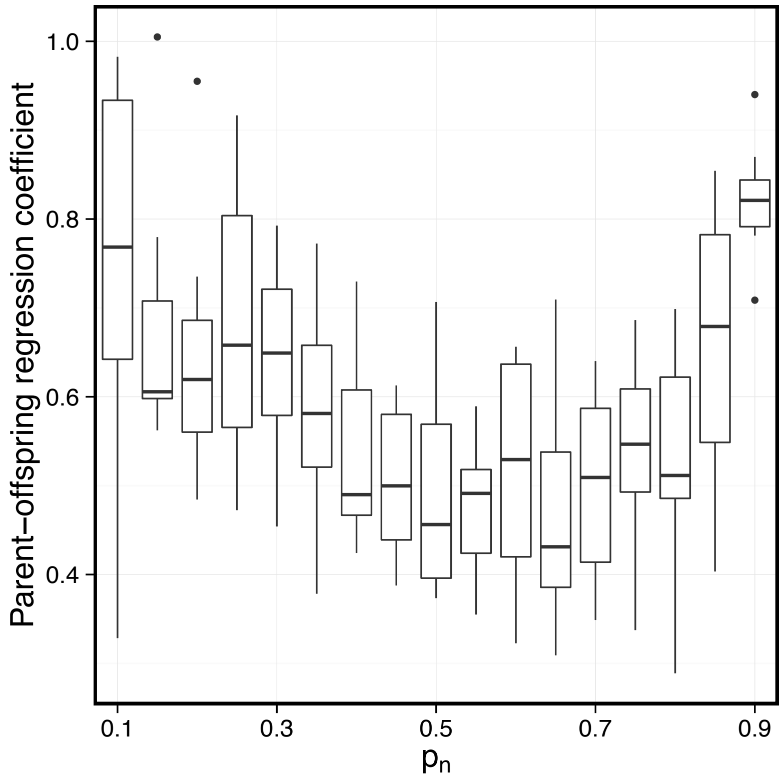

Supplementary Figure 13: **The heritability of local clustering coefficients.** The figure depicts the regression of clustering coefficients among parents and their offspring as a function of the strength of social inheritance (p_n). The bottom and top of the box mark the first and third quartiles. The upper whisker extends from the hinge to the highest value that is within 1.5*IQR of the hinge, where IQR is the inter-quartile range, or distance between the first and third quartiles. The lower whisker extends from the hinge to the lowest value within 1.5*IQR of the hinge. Data beyond the end of the whiskers are outliers and plotted as points. Ten replicate simulations were run for each p_n value. Parameter values: simulation steps=2000 (parent-offspring regression calculated for the last 100 offspring born), $N = 100$, $p_r = 0.01$.

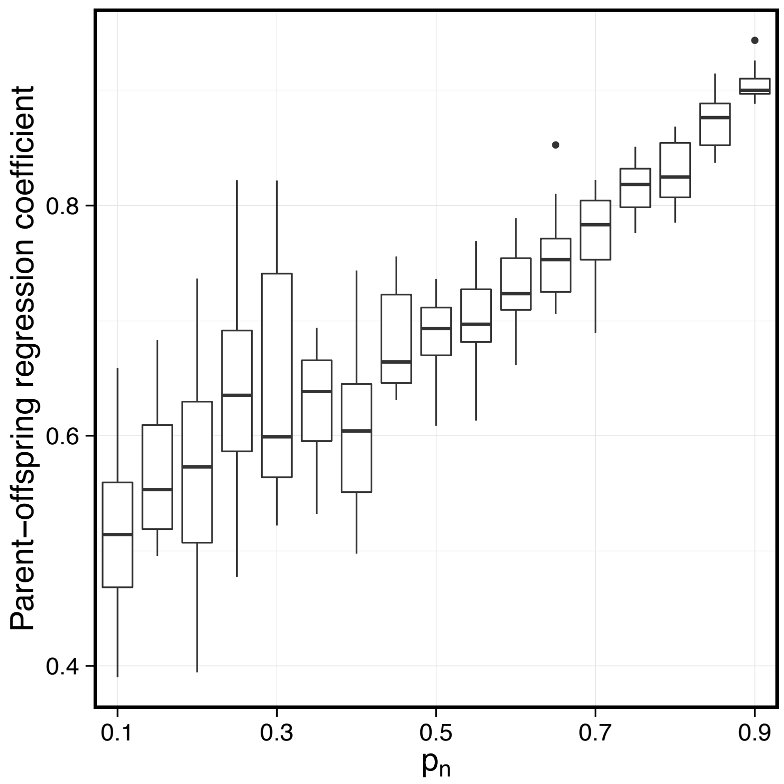

Supplementary Figure 14: **The heritability of eigenvector centrality.** The figure depicts regression of eigenvector centrality among parents and their offspring as a function of the strength of social inheritance (p_n). The bottom and top of the box mark the first and third quartiles. The upper whisker extends from the hinge to the highest value that is within 1.5*IQR of the hinge, where IQR is the inter-quartile range, or distance between the first and third quartiles. The lower whisker extends from the hinge to the lowest value within 1.5*IQR of the hinge. Data beyond the end of the whiskers are outliers and plotted as points. Ten replications were run for each p_n value. Parameter values: simulation steps=2000 (parent-offspring regression calculated for the last 100 offspring born), $N = 100$, $p_r = 0.01$.

Supplementary Tables

Species	p_n	p_r
Spotted hyena	0.86	0.021
Rock hyrax	0.76	0.01
Bottlenose dolphin	0.50	0.033
Sleepy lizard	0.53	0.003

Supplementary Table 1: Parameter values used in the simulations of the two-sex model for each species in Supplementary Figure **8**.
